# Radiological changes in shoulder osteoarthritis and pain sensation correlate with patients’ age

**DOI:** 10.1186/s13018-022-03137-x

**Published:** 2022-05-15

**Authors:** Nicole Märtens, Vincent März, Jessica Bertrand, Christoph H. Lohmann, Alexander Berth

**Affiliations:** 1 Department of Orthopaedic Surgery, Otto-Von-Guericke-University, Magdeburg, Germany; 2 Departement of Orthopaedic Surgery, Otto-Von-Guericke Universität, Leipziger Str. 44, 39120 Magdeburg, Germany

**Keywords:** Osteoarthritis, Shoulder, OARSI, Constant–Murley score, Age, Pain

## Abstract

**Purpose:**

Osteoarthritis (OA) is one of the most common musculoskeletal disorders in the aging population. The correlation of radiographic OA severity, disability and pain is variable and inconsistent for the different joints. This study aims to elucidate the relationship between histological and radiological signs of shoulder OA with pain sensation and functional impairment to potentially adapt the recommendation for surgical treatment for primary total shoulder arthroplasty (TSA).

**Methods:**

Forty-four patients with shoulder OA undergoing TSA using an anatomic stemless implant were included in this study. The radiological OA severity was scored pre-operatively on true ap X-rays according to the Kellgren–Lawrence score (KL-Score). Acromial types according to Bigliani were defined by pre-operative radiological images. The histological OA severity was determined according to the OARSI-Score using bone–cartilage sections from loaded areas of the humeral head. Pain was quantified using the visual analog scale (VAS). The functional status was assessed by the items “mobility” and “strength” out of the Constant–Murley score (CS Score). Demographic data including BMI, age, gender, diabetes mellitus and smoking were recorded.

**Results:**

There was no correlation between radiographic and histological severity in shoulder OA. However, a correlation of age and the severity of radiological changes was observed. Further, pain did not correlate with histological or radiological scores, whereas it correlated with age and the presence of diabetes mellitus. The functional shoulder status (mobility, strength) correlated with the severity of radiological changes, but not with the histologic scoring, which correlated with nicotine abuse.

**Conclusion:**

This study shows that increased age is the main determinant of radiological changes in shoulder OA, as well as pain. Therefore, age and pain sensation should be considered as important parameters for the recommendation for TSA.

**Supplementary Information:**

The online version contains supplementary material available at 10.1186/s13018-022-03137-x.

## Background

Osteoarthritis (OA) is one of the most common age-related diseases [1]. OA is characterized by progressive loss of cartilage, new bone formation and inflammation of the synovial tissue [2]. Clinically, OA can be diagnosed by the radiographic evidence of cartilage damage and it is considered to cause increased complaints about joint pain, restricted activities of daily living, loss of function and strength of the affected joint [3]. The Kellgren–Lawrence score (KL- Score), therefore, has been proposed as one radiographic diagnostic method to estimate the extent of shoulder OA [4]. Therefore, OA can also be approached by assessing subjective and objective determinants of the respective joint.

A correlation between structural damage and radiological appearance for hip and knee OA has been clearly shown previously [5, 6]. Additionally, some studies showed a correlation between pain and radiological OA severity [7, 8]. This correlation, however, is controversially discussed in the literature for both, the knee and the hip joint [9, 10]. One study showed that an increased radiological score in the knee joint was associated with increased pain frequency scores [11]. Another study showed that no significant association between radiographic lesions and pain frequency could be proven for the hip, giving rise to the assumption that the pain sensation might be joint specific [12].

OA of the shoulder joint is less frequent than hip or knee OA [2, 3]. Furthermore, there is even less information about the correlation between radiographic changes and pain for the shoulder joint. The optimal management of primary shoulder OA is a topic of ongoing investigation [13]. Beside conservative treatment, numerous surgical techniques exist including non-arthroplasty techniques and anatomic or reversed shoulder replacement systems [13]. Although primary total shoulder arthroplasty (TSA) has been accepted as an effective and secure procedure to relieve pain and to restore shoulder function in patients with end-stage OA, who failed to conservative treatment, it is potentially associated with a number of complications [14–16]. Therefore, the indication for TSA needs to be proven carefully. Besides a justified indication for TSA considering the respective joint pathology, an accurate surgical technique with utilization of an adequate implant and the appropriate patient selection are essential factors for a successful outcome of TSA.

The aim of this study was to elucidate the relation between radiological changes and histological appearance of OA with pain sensation as well as the functional status in patients with shoulder OA. This aims to provide more insight to amend the recommendations for TSA in patients with shoulder OA.

## Methods

### Patients

This study included 44 patients (19 male/25 female, 68.5 ± 17.5 years) with primary OA of the shoulder treated with a stemless humeral head replacement between December 2011 and May 2013. Informed consent obtained from all patients enrolled in the study after IRB approval of the faculty of medicine (IRB No.: 55/11). The number of previous surgeries or arthroscopies at the respective shoulder joint for each patient were assessed. During surgery, we examined the condition of the tendons in the shoulder, as well as the rotator cuff. Ten patients underwent previous arthroscopic surgery (n = 8 subacromial decompression, n = 2 subacromial decompression with rotator cuff repair). We did not include any patient with secondary OA due to chronic instability or patients with a cuff tear arthropathy. Demographic data such as age, gender, BMI, smoking and number of previous surgeries at the respective shoulder were recorded and are listed in Additional file 2: Table S2.

### Radiographic analysis

Standardized X-ray images in true anterior–posterior, outlet and axial view were performed pre-operatively. The KL-Score was used for scoring of shoulder OA severity [4]. As we did not include instability of the shoulder joint as well as rotator cuff arthropathy in our study, we decided to use the KL-Score instead of the Samilson or Hamada–Fukuda score. The KL-Score is mainly based on the quantification of osteophytes, joint space narrowing and subchondral bone sclerosis. The glenoid morphology was classified according to Walch et al. in axial view X-ray [17] Additional file 3. The acromial types according to Bigliani were scored in outlet view X-ray [18]. All radiographs were reviewed by two independent observers which were 2 experienced shoulder surgeons, and a consensus was achieved. 43 out 44 X-ray sets could be examined, except that one X-ray cannot be assessed due to incorrect radiographic adjustment.

### Histological analysis

OA grade was evaluated using a histology score (OARSI-Score). Briefly, the resected humeral head was cut into quarter slices (centre to periphery). The humeral histological scoring represents the osteoarthritis grade of the central, main load bearing, cartilage area of the humeral head. All specimens were fixed in 4% PFA for 24 h, decalcified and embedded in paraffin. 3–4 µm sections were cut and stained using Safranin Orange. OARSI-Score was used to assess the histological osteoarthritis score (grades 0–6) [19]. The classification of OARSI scoring for each blinded sample, which was reviewed by two independent observers, and a consensus score was generated during a meeting.

### Clinical evaluation

One of the authors clinically examined all patients before surgery. The functional status of the patients was assessed with the items “mobility” and “strength” according to the Constant–Murley score [20]. The pain quantification in the Constant–Murley score describes the average pain level of each patient during one week of regular movement, as described by the VAS pain questionnaire. Thus, the pain sensation of each patient was assessed using a visual analog scale (VAS; 0—no pain, 15—max. pain). However, the Constant–Murley score does not evaluate the acute pain during a specific movement or mobilization. Pre-operatively, the following rotator cuff tests were performed for each patient: Jobe test for supraspinatus muscle, lift-off and belly press test for subscapularis muscle and external rotation lag sign for assessing the infraspinatus muscle. Comorbidities (smoke; metabolic diseases—diabetes) and BMI are all recorded in an anaesthetic protocol. We classified the satisfaction with the shoulder replacement of each individual patient outcome after 8–12 month using a questionnaire giving four choices: very good (1), good (2), satisfactory (3) and bad (4). Furthermore, the range of motion was evaluated after this time.

### Statistical analysis

Statistical analysis performed using GraphPad 6 Software (La Jolla California, USA). The statistical significance was analysed using a linear regression. The Pearson’s coefficient r^2^ is given as indicator for correlation between both tested parameters. Statistical significance was considered for a p value < 0.05.

## Results

### Description of the patient cohort

The intraoperative assessment of the rotator cuff shows a chronic tendinopathy (fraying and fragmentation of rotator cuff fibres and partial supraspinatus tendon tears) in six patients, whereas the periarticular tendons (conjoint tendons, pectoralis major/minor) show no pathologies. The pre-operative functional joint status of the patients was measured using the Constant–Murley (CS) score with an average of 13.2 ± 7.4 points for “mobility” and 2.6 ± 2.3 points for “strength” in the total cohort. The pre-operative pain sensation of the patients was measured using the VAS-Pain score (8.7 ± 2.6 points). When comparing the CS-Score data for all included patients, we did not observe an influence of the observed rotator cuff tendinopathy in the six included patients on the obtained values. For this reason, we assumed that the rotator cuff tendinopathy does not affect the evaluated CS-Score items. The radiographic evaluation of the true anteroposterior X-rays showed a grade 1 in 5 patients, a grade 2 in 10 patients, a grade 3 in 6 patients and a grade 4 in 23 patients according to the KL-Score. Evaluation of the acromiohumeral distance shows no presence of superior migration of the humeral head in the total cohort. We further compared the six patients with the rotator cuff tendinopathy with the other patients from the cohort and did not observe a specific increase in KL-Score, which could have been attributed to the tendon condition. Therefore, we decided to include the six patients in our complete cohort.

### No correlation between radiological and histological OA grade

**Fig. 1:**
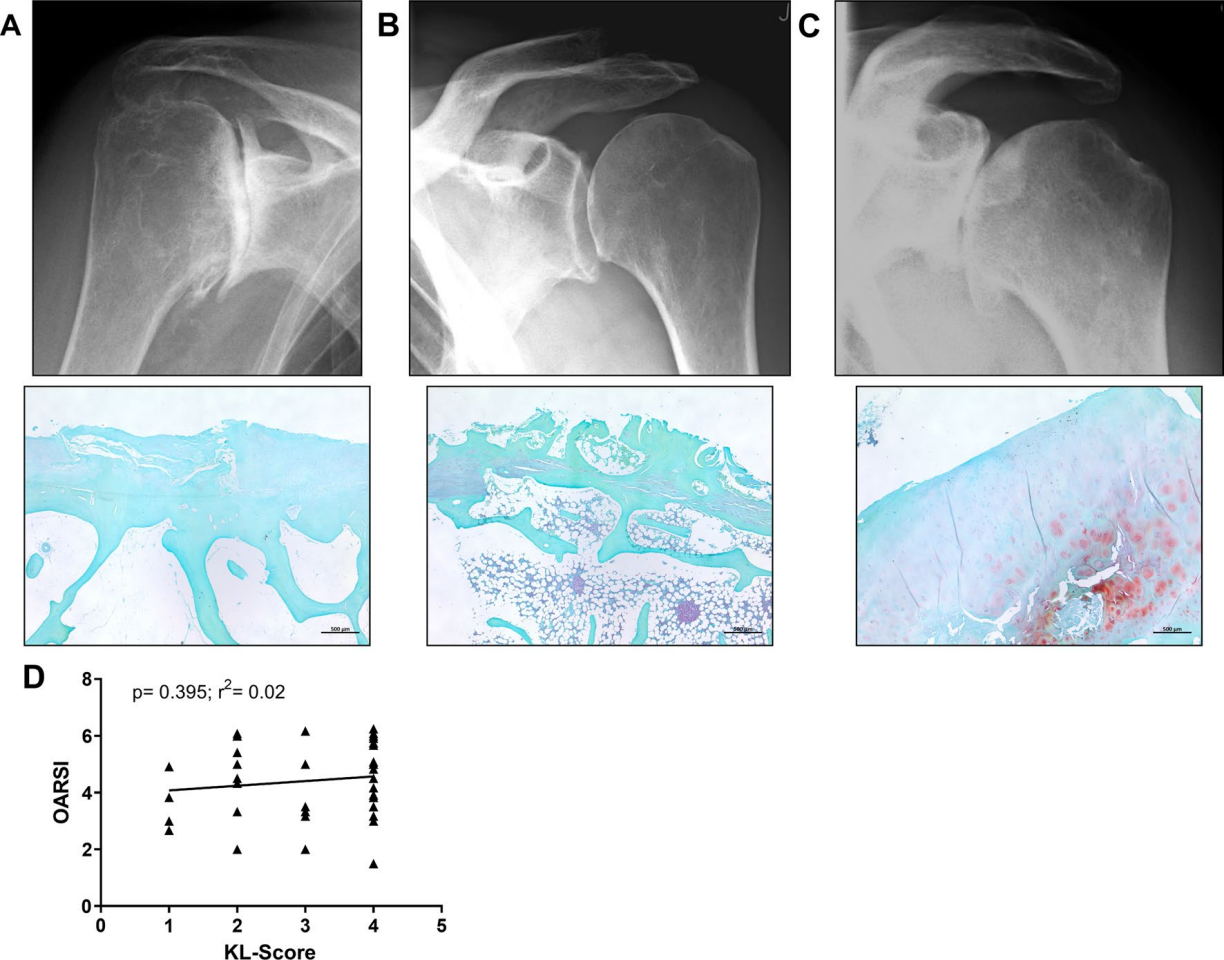
No correlation between radiological and histological OA grade. **A** Representative true anteroposterior X-ray picture with Kellgren–Lawrence grade 4 and corresponding histological cartilage evaluation (OARSI 5). **B** True anteroposterior X-ray picture with KL grade 2 and corresponding histology (OARSI 6). **C** True anteroposterior X-ray with KL grade 4 and corresponding histology (OARSI 4) **D** Correlation between OARSI-Score

Additionally, we scored cartilage–bone sections from the main loaded area of the humeral head using the OARSI-Score. When comparing the radiographic OA severity with histological signs of OA, there were patients with severe radiographic and histological OA (Fig. 1a). Interestingly, we also observed patients with low radiographic OA severity, but marked histological OA severity (Fig. 1b). However, we also found patients with severe radiographic OA and mild OA like cartilage changes (Fig. 1c). The classification of the histological OA grade of the specimens according to the OARSI-Score demonstrates grade 1 in 1 patient, a grade 2 in 3 patients, a grade 3 in 13 patients and a grade 4 in 6 patients, grade 5 in 11 patients and grade 6 in 7 patients. In line with the above mentioned observations, we found no correlation in the anterior–posterior projection in KL classification and OARSI scoring for comparing the grade of shoulder OA (p = 0.4) (Fig. 1d). When comparing the OARSI-Score with smoking, we observed a correlation between both factors (p* < 0.047; r^2^ = 0.3). No correlation was found when comparing the OARSI-Score with the presence of diabetes mellitus for each patient (p < 0.49, r^2^ = 0.1) and between OARSI and BMI (p < 0.9408, r^2^ = 0.01) (Additional file 5: Table S5). Gender distribution among BMI (p < 0.658) and diabetes mellitus (p < 0.5172) was not significant (data not shown).

### Correlation between pain and OA severity

**Fig. 2:**
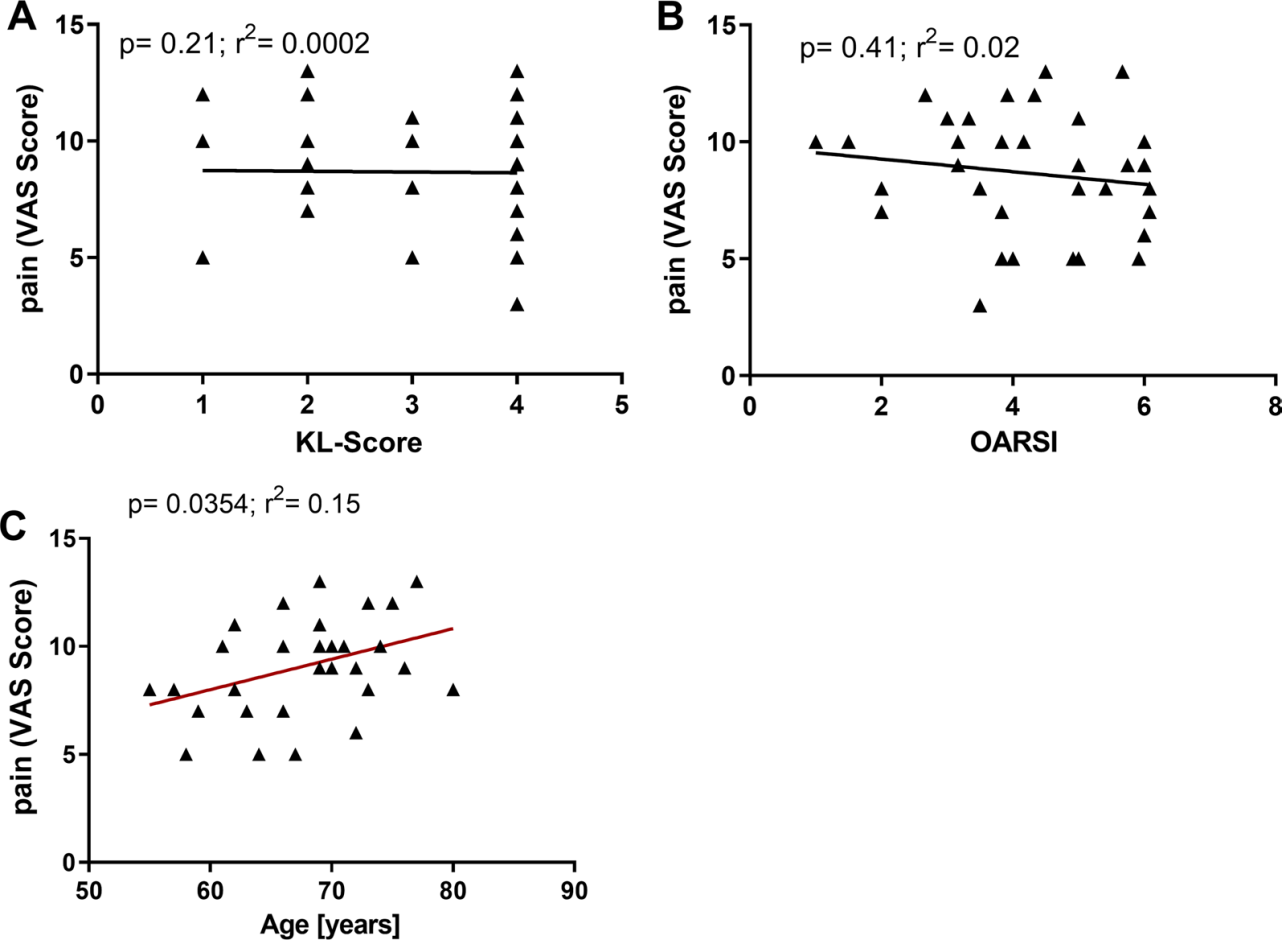
No correlation between radiological and histological OA grade. **A** Linear regression analysis of pain (VAS-Score) data with Kellgren–Lawrence score (N = 33, p = 0.21). **B** Linear regression analysis of pain (VAS-Score) with OARSI-Score (N = 31, p = 0.41). **C** Liner regression analysis of pain (VAS-Score) with age (N = 31, p = 0.0354)

To understand whether there is a correlation between pain and the radiographic OA severity, the KL-Score was plotted against the values from the VAS-Pain-Scale specific for the shoulder for each patient. There was no significant correlation between KL-Score and VAS-Pain-Scale (p = 0.2) (Fig. 2a). Furthermore, there was no correlation between the VAS-Pain-Scale and the OARSI-Score (p = 0.4) (Fig. 2b) in the tested cohort. Additionally, the different acromial types did not correlate with the VAS-Pain-Scale (p < 0.3; r^2^ = 0.2) (Additional file 5: Table S5). When comparing the pain reception for each patient with the presence of diabetes mellitus, we found a correlation between both (p** < 0.005, r^2^ = 0.47) (Additional file 5: Table S5). Interestingly, a correlation was found when comparing age with pain (p = 0.04; r^2^ = 0.15) (Fig. 2c) for each patient. However, age did not correlate with the presence of diabetes mellitus (p < 0.2; r^2^ = 0,2) (Additional file 5: Table S5).

### Joint function correlated with KL-score, but not with histological assessment

**Fig. 3:**
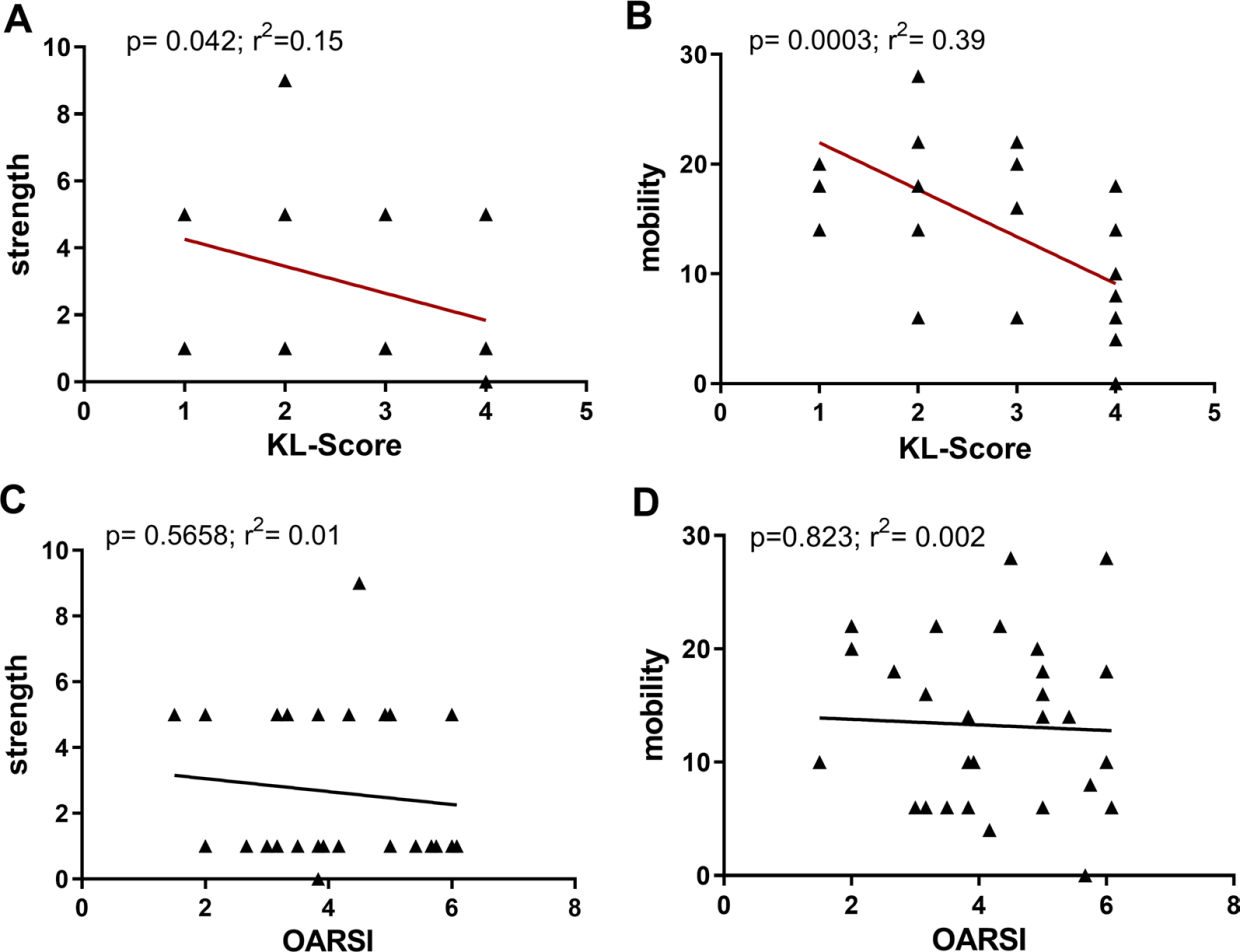
Joint function correlated with KL-Score, but not histological assessment. **A** Linear regression analysis of the Constant–Murley score item “strength” and “mobility” (**B**) with radiological changes (N = 31, p = 0.042 and p = 0.003, respectively. **C** Linear regression analysis of the Constant–Murley score item “strength” and “mobility” (**D**) with the histological OARSI-Score (N = 31, p = 0.5558 and 0.823, respectively)

Furthermore, we tested the influence of radiological and histological signs of OA on the joint function. The correlation analysis between the KL-Score and the functional status of the shoulder joint, represented via the CS-Score items “mobility” and “strength”, was significant (p = 0.0003; r^2^ = 0.39 and p = 0.04; r^2^ = 0.15, respectively) (Fig. 3a, b). However, “mobility” and “strength” did not correlate with the histological OARSI-Score (p = 0.6 and p = 0.8, respectively) (Fig. 3c, d).

### Age correlated with Kellgren–Lawrence score, but not OARSI-score

**Fig. 4:**
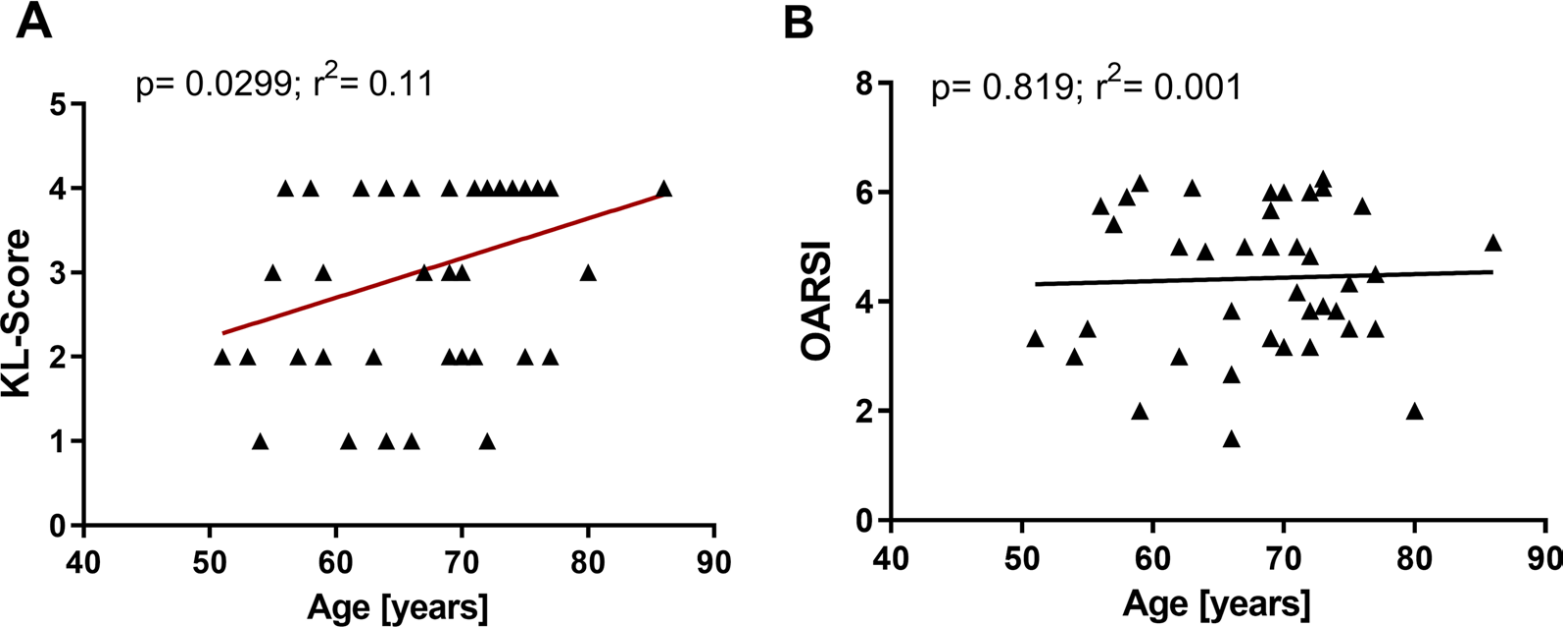
Age correlated with Kellgren–Lawrence score, but not OARSI-Score. **A** KL-Score correlation with age (N = 44, p = 0.0299) **B** OARSI-Score correlation with age (N = 41, p = 0.819)

Since OA was proposed to be an age-related disease, we investigated the correlation between radiological signs of OA using the KL-Score and age. We observed a correlation between both parameter (p = 0.03; r^2^ = 0.11) (Fig. 4a). A correlation analysis between the histological OA grade and the age of the respective patient revealed no correlation between both parameters (p = 0.8) (Fig. 4b). Determining acromial type according to Bigliani showed acromial Type 1 in 25 cases, acromial type 2 in 17 cases and acromial type 3 in 1 case. There was no correlation of Bigliani type or pain (p < 0.2963) or OARSI-Score (p < 0.6784) (Additional file 4 and 5: tables S4 + S5).

### Patients’ outcome

After a time period of about one year, we neither found a correlation in post-operative range of motion with pre-operative OA severity (KL-Score) (p < 0.06) as well as with the pre-operative histological OA severity (OARSI-Score) (p < 0.99). However, we observed a positive correlation of the pre-operative radiological OA severity and post-operative pain p < 0.048 (N = 27).

## Discussion

The aim of the study was to investigate the interaction between typical clinical findings in patients with shoulder OA who failed to conservative treatment in consideration to the indication of primary TSA. Therefore, correlation analysis performed including the parameters pain, BMI, diabetes mellitus, smoking, functional involvement, radiological and histological appearance and age in patients undergoing a stemless humeral head replacement. The outcome parameters were evaluated for post-operative pain and range of motion that were correlated with the pre-operative radiological and histological severity. To our knowledge, this is the first study describing the relationship between age, pain, diabetes mellitus as well as radiographic scoring in patients with osteoarthritis of the shoulder. We observed that histological degradation processes correlate with nicotine but not with pain, age and KL-Score in patients with shoulder OA.

The findings of the present study lead to the assumption that pain sensation correlates with patient age and diabetes mellitus. Furthermore, our data show a correlation between severity of radiographic changes of arthritic shoulder joint and the functional impairment of the involved shoulder. The characteristic histologic evaluation of structural cartilage damage in shoulder OA does not seem to be directly associated with the radiographic severity as well with the limitations in function and pain sensation of the affected arthritic joint but with nicotine (overview in Additional file 1 and 5: tables S1, S5). The outcome evaluation could be influenced by several parameters, and this study might give insight in the proposed success rate for patient’s satisfaction with different OA stages and radiological OA changes.

The success of TSA as a treatment option in patients with shoulder OA is depending on many factors [21]. Objective parameters such as the improvement of range of motion and an increase of strength as well as subjective values like pain reduction are involved in patient satisfaction after surgery [22, 23]. The observation that the radiographic OA signs increase with increasing patient age is in line with a previous study [24]. However, the missing correlation between radiographic OA changes and histological OA changes has not been described so far. To our knowledge, only one study has analysed the relationship of signs of OA, e.g. the hyaline articular cartilage calcification with age and histologic OA grade using the OARSI-Score before [25]. In line with our findings, the authors did not observe a correlation between OARSI-Score and age. However, the study did not include the KL-Score or the VAS-Pain-Score of the patients since this study included only post-mortem samples. The phenomenon that the histological OARSI-Score correlates with the radiographic changes has been described for hand and knee OA before [5, 6]. A possible explanation for this discrepancy in the shoulder joint might be due to limitations of grading osteochondral lesions with X-ray radiographs utilizing the KL-Score, which was not originally applied to the glenohumeral joint [4]. Furthermore, shoulder OA is often associated with a posterior humeral head subluxation. Therefore, it is possible that the size and location of resulting osteochondral lesions (“posterior wear”) and osteophytes may be not fully estimated using X-ray analyses [26]. The use of ultrasound could be an additional instrument for recording osteochondral lesions in humeral head and thereby might be an additional tool in the investigation of shoulder OA, as it has already been established in the knee [27, 28].

The data of the present study suggest that neither the radiographic nor the histological joint changes had a significant influence on the pain sensation in patients with OA. This observation is in line with a previous study also showing that the radiographic changes in the shoulder do not correlate with the VAS-Pain-Score [29]. Indeed metabolic diseases such as diabetes had an impact on the individual pain reception in our cohort. An influence of diabetes mellitus on pain reception has already been shown in another study for knee OA [30]. In contrast, other studies show a correlation between pain intensity and KL-Score for other joints, e.g. the knee [7, 31] or hip [8]. These incongruent results within several joints lead to the assumption that pain intensity and disease severity in radiographs may differ for each joint of the body. This discrepancy may also be explained by some aspects of the pathology of OA that are related to pain like synovitis or bone marrow oedema that cannot be seen in X-ray imaging [32]. Additionally, periarticular pathologies such as subacromial bursitis and/or rotator cuff—and biceps tendinitis, are also common in patients with shoulder OA resulting in local pain and not depicted in X-ray pictures. These numerous “non-articular pain conditions” might also contribute to the discrepancy between pain intensity and the radiographic severity in the shoulder OA [32].

The average age in this study population is in the typical range for OA [33]. In this context, the present study shows that patient age and diabetes mellitus correlated with pain intensity estimated by a VAS-Score. This finding indicates that age-related effects might influence pain sensation in shoulder OA. Several studies have evaluated the impact of age as a risk factor for pain perception [34–36]. Interestingly, animal studies demonstrated that the sensitization of nociceptors to mechanical stimulation is dependent on age and the chronicity of the inflammation [35]. Moreover, human OA pain sensation is a complex phenomenon and beside structural articular pathologies like cartilage alterations or inflammation due to synovitis it is caused and modulated by various other factors, e.g. central pain pathways and genetic factors [37–39].

This study also shows that the functional status of the shoulder, evaluated according to the CS-Score items “mobility” and “strength”, is clearly associated with the severity of the radiographic articular changes. This might be explained by the fact that a decreased joint volume, a tight capsule, osteophytes and the deformation of the articular surface may cause a mechanical restriction of the joint motion as well as a reduced ability to raise and hold a specific weight in a given position. However, the histopathological evidence of glenohumeral cartilage degeneration did not seem to affect shoulder function and pain sensation as shown in this present study. It is well known that in contrast to osteochondral defects of weight-bearing joints such as the hip, knee and ankle, even large degenerative glenohumeral cartilaginous lesions can be well tolerated [40]. As the shoulder is a non-weight-bearing joint, a strong correlation between the extent of cartilage damage, pain intensity and functional disability is lacking.

We are well aware of the limitation of our study which includes only 44 patients. More patients are needed to unravel the complex relations between structural joint damage and pain sensation. Furthermore, we investigated the range of motion of the shoulder joints in the present cohort using the Constant–Murley score assessing the active mobility of the shoulder; passive motion is not described in the score. The findings of the physical examination of the patients in our study findings demonstrate a comparable restricted active and passive range of motion in all planes of movement. Further, we did not investigate the pain due to muscle contraction in our study. We assume that the patients might have suffered from increased muscle tension and compensation movement due to their shoulder pain, which was not included in our pain analyses. Although our results did not show an influence of the acromial type on the pain assessment of our patients, we could not exclude that the shape of the acromial arch, the glenoid morphology or rotator cuff pathologies may influence shoulder pain and may influence or affect the pain sensation in the individual patient. A limitation of the study is the use of humeral head only, giving only a partial view of the total shoulder joint cartilage, which might have influenced the correlation with radiological KL-Score as well as pain. For evaluating a potential influence of comorbidities (such as diabetes mellitus) or environmental factors (such as smoking) on the onset of shoulder OA or pain reception of the patient, a larger patient cohort will be needed to draw conclusions.

## Conclusion

The diagnosis and treatment options of shoulder OA are commonly based on functional impairment and radiographic signs of OA. This study shows that particularly pain intensity and patient age are important parameters in supporting treatment plans for shoulder OA and should be taken into account for decision making in TSA. However, further investigations are needed to evaluate the exact impact of the structural pathology, functional limitations and pain for the recommendation of TSA in patients with shoulder OA.

## Supplementary Information

**Additional file 1: Table S1.** Summary of correlation analysis. The KL-Score shows a positive correlation with age, shoulder function (mobility, strength) and patients’ outcome. The VAS-Pain-Score shows a positive correlation with age.

**Additional file 2: Table S2.** Summary of patient cohort. The number of patients according to age, gender, BMI, number of diabetes mellitus, smoking and previous surgeries.

**Additional file 3: Table S3.** Summary of glenoid morphology. The number of patients according to the different types of glenoid formation (Walch)

**Additional file 4: Table S4.** Summary of acromial types. The number of patients according to number of different acromial types (Bigliani 1–3)

**Additional file 5: Table S5.** Summary of correlation analysis. The OARSI score show a positive correlation with smoke. The VAS- Pain- Score shows a positive correlation with diabetes mellitus.

## Data Availability

The data that support the findings of this study are available from the authors. Data are, however, available from the authors upon reasonable request.

## Abbreviations

TSA: Total shoulder arthroplasty
OA: Osteoarthritis
CS: Constant–Murley score
KL-Score: Kellgren and Lawrence score
OARSI-Score: Histology score
VAS pain score: Visual analog scale pain score
BMI: Body mass index
