# Supplementary material for: Radiological changes in shoulder osteoarthritis and pain sensation correlate with patients’ age
Source: J Orthop Surg Res. 2022 May 15;17:277. doi: 10.1186/s13018-022-03137-x (PMC9107673; doi:10.1186/s13018-022-03137-x)
Supplement: Supplementary file 1 — Additional file 1: Table S1. Summary of correlation analysis. The KL-Score shows a positive correlation with age, shoulder function (mobility, strength) and patients’ outcome. The VAS-Pain-Score shows a positive correlation with age. [file 13018_2022_3137_MOESM1_ESM.docx]

Table 1: Summary of correlation analysis.

|  | KL-Score | OARSI-Score | VAS-Pain-Score | Age | Mobility | Strength |
| --- | --- | --- | --- | --- | --- | --- |
| KL-Score |  |  |  | X | X | X |
| OARSI-Score |  |  |  |  |  |  |
| VAS-Pain-Score |  |  |  | X |  |  |
| Age | X |  | X |  |  |  |
| Mobility | X |  |  |  |  |  |
| Strength | X |  |  |  |  |  |
| Outcome pain | X |  |  |  |  |  |
| Outcome mobility |  |  |  |  |  |  |
