# Supplementary material for: Radiological changes in shoulder osteoarthritis and pain sensation correlate with patients’ age
Source: J Orthop Surg Res. 2022 May 15;17:277. doi: 10.1186/s13018-022-03137-x (PMC9107673; doi:10.1186/s13018-022-03137-x)
Supplement: Supplementary file 2 — Additional file 2: Table S2. Summary of patient cohort. The number of patients according to age, gender, BMI, number of diabetes mellitus, smoking and previous surgeries. [file 13018_2022_3137_MOESM2_ESM.docx]

Table 2: Summary of patient cohort

| Patient cohort | number of patients |
| --- | --- |
| number of patient | 44 |
| age | 62,5 years |
| gender | 26f/18m |
| diabetes | 10 |
| nicotine | 5 |
| BMI | 28,86+-4,343 |
| surgery | 10 |
