# Supplementary material for: Radiological changes in shoulder osteoarthritis and pain sensation correlate with patients’ age
Source: J Orthop Surg Res. 2022 May 15;17:277. doi: 10.1186/s13018-022-03137-x (PMC9107673; doi:10.1186/s13018-022-03137-x)
Supplement: Supplementary file 3 — Additional file 3: Table S3. Summary of glenoid morphology. The number of patients according to the different types of glenoid formation (Walch) [file 13018_2022_3137_MOESM3_ESM.docx]

Table 3: Summary of glenoid morphology

| Glenoid formation  Walch | A1 | A2 | B1 | B2 | C |
| --- | --- | --- | --- | --- | --- |
| Number of patients 44 | 26 | 6 | 11 | 1 | 0 |
