# Supplementary material for: Radiological changes in shoulder osteoarthritis and pain sensation correlate with patients’ age
Source: J Orthop Surg Res. 2022 May 15;17:277. doi: 10.1186/s13018-022-03137-x (PMC9107673; doi:10.1186/s13018-022-03137-x)
Supplement: Supplementary file 4 — Additional file 4: Table S4. Summary of acromial types. The number of patients according to number of different acromial types (Bigliani 1–3) [file 13018_2022_3137_MOESM4_ESM.docx]

Table 4: Summary of acromial types in our cohort

| Patient cohort | Acromial  Type 1 | Acromial  Type 2 | Acromial  Type 3 |
| --- | --- | --- | --- |
| Number of patients 43 | 25 | 17 | 1 |
