# Supplementary material for: Radiological changes in shoulder osteoarthritis and pain sensation correlate with patients’ age
Source: J Orthop Surg Res. 2022 May 15;17:277. doi: 10.1186/s13018-022-03137-x (PMC9107673; doi:10.1186/s13018-022-03137-x)
Supplement: Supplementary file 5 — Additional file 5: Table S5. Summary of correlation analysis. The OARSI score show a positive correlation with smoke. The VAS- Pain- Score shows a positive correlation with diabetes mellitus. [file 13018_2022_3137_MOESM5_ESM.docx]

Table 5: Summary of correlation analysis.

|  | pain | OARSI | Smoke | Diabetes | BMI |
| --- | --- | --- | --- | --- | --- |
| pain |  | - | - | ++ | - |
| OARSI | - |  | + | - | - |
| smoke | - | + |  | - | - |
| diabetes | ++ | - | - |  | - |
| BMI | - | - | - | - |  |
| Acromialtype | - |  |  |  |  |

Pain/diabetes p**; p<0,0051

OARSI/smoke p*; p<0,0469
